# Atypical Legionella pneumophila encephalopathy lacking respiratory symptoms and radiographic lesions: A Case Report

**DOI:** 10.3389/fmed.2026.1828042

**Published:** 2026-05-20

**Authors:** Weiwei Yan, Xi Wang, Kening Shi, Luye Wang

**Affiliations:** Department of Critical Care Medicine, Weifang People's Hospital, Weifang, Shandong Province, China

**Keywords:** atypical presentation, encephalopathy, glucocorticoids, Legionella pneumophila, targeted next-generation sequencing

## Abstract

This report details an unusual case of Legionella pneumophila encephalopathy in a 29-year-old male who presented with acute altered consciousness and extreme agitation, notably lacking any respiratory symptoms or typical meningeal signs. Extensive imaging, including chest CT and cranial MRI, revealed no pulmonary infiltrates or structural brain lesions. Cerebrospinal fluid (CSF) analysis demonstrated an aseptic profile with elevated protein, and CSF metagenomic sequencing returned negative. The diagnostic dilemma was ultimately resolved using whole-blood targeted next-generation sequencing (tNGS), which detected Legionella sequences. The patient achieved a rapid and complete neurological recovery following a combined regimen of levofloxacin and high-dose glucocorticoids. This case underscores that Legionella infection can manifest as an isolated, toxin- and immune-mediated encephalopathy without preceding clinical pneumonia. It highlights the critical rescue value of early molecular screening (such as tNGS) in unexplained encephalopathy and supports the judicious use of early steroid intervention to halt the aseptic neurotoxic cascade.

## Introduction

Legionella, predominantly Legionella pneumophila, is a Gram-negative, aerobic intracellular parasite commonly found in natural freshwater ecosystems and man-made water supply systems, including cooling towers, hot water circulation systems, air conditioning units, and public shower facilities (1, 2). The bacterium predominantly induces infection via human inhalation of contaminated aerosols harboring the pathogen (3). In recent years, with the intensification of global climate change, population aging, and the continuous increase in immunocompromised individuals, the global incidence of Legionella infection has shown a significant upward trend, becoming one of the infectious diseases that seriously threaten global public health security (4). Traditional views hold that Legionella infection primarily manifests as pulmonary infiltrates and severe respiratory failure (1). However, numerous clinical cohort studies and retrospective analyses indicate that Legionella infection is characterized by significant multisystem and extrapulmonary organ involvement, with central nervous system (CNS) involvement being the most prominent and highly lethal (5). Among patients in the acute phase of Legionella infection, as many as 50% develop neurological abnormalities of varying severity during the course of the disease (5). These abnormalities are collectively termed “Legionella encephalopathy” (5). The clinical presentation of Legionnaires' disease exhibits high heterogeneity, with symptoms ranging from mild attention deficits and confusion to severe delirium, focal neurological deficits, and deep coma. Clinicians should be highly alert to the fact that Legionella encephalitis often presents with nonspecific clinical manifestations, and standard imaging studies (such as cranial CT and conventional MRI sequences) frequently fail to reveal significant structural lesions. Furthermore, cerebrospinal fluid (CSF) analysis in most patients yields “aseptic” results—whether assessed through routine biochemistry, cell counts, pathogen cultures, or PCR testing—particularly in those without typical Legionella lower respiratory tract infection symptoms. This poses significant challenges for the early identification and confirmation of Legionella meningoencephalitis (6).

This report describes a case of acute Legionella encephalitis in a young male presenting without typical respiratory symptoms and negative imaging findings, aiming to heighten clinicians' awareness of the atypical manifestations of this disease.

## Case presentation

The patient is a 29-year-old male residing in an urban area. He works as an office employee, denying any specific occupational hazards related to soil, industrial cooling towers, or contaminated public water sources. He also reported no recent travel, tourist routes, or hotel accommodations within the month prior to symptom onset. Notably, due to the dry indoor environment caused by winter central heating, he regularly used a domestic air humidifier at home, which represents a highly plausible epidemiological source for aerosolized Legionella exposure. Regarding his social history, he is a non-smoker and consumes alcohol only occasionally in social settings.

His past medical history was notable only for elevated fasting blood glucose levels discovered 3 months prior to admission during a routine annual physical examination organized by his employer; however, he did not undergo any medication-based hypoglycemic treatment. He had no history of chronic diseases, long-term medication use, or prior glucocorticoid or immunosuppressant treatments. His baseline immune status was competent, with no history of HIV infection, organ transplantation, or recent surgical interventions. Furthermore, he denied any other recent infections prior to the onset of the current illness.

**Figure 1:**
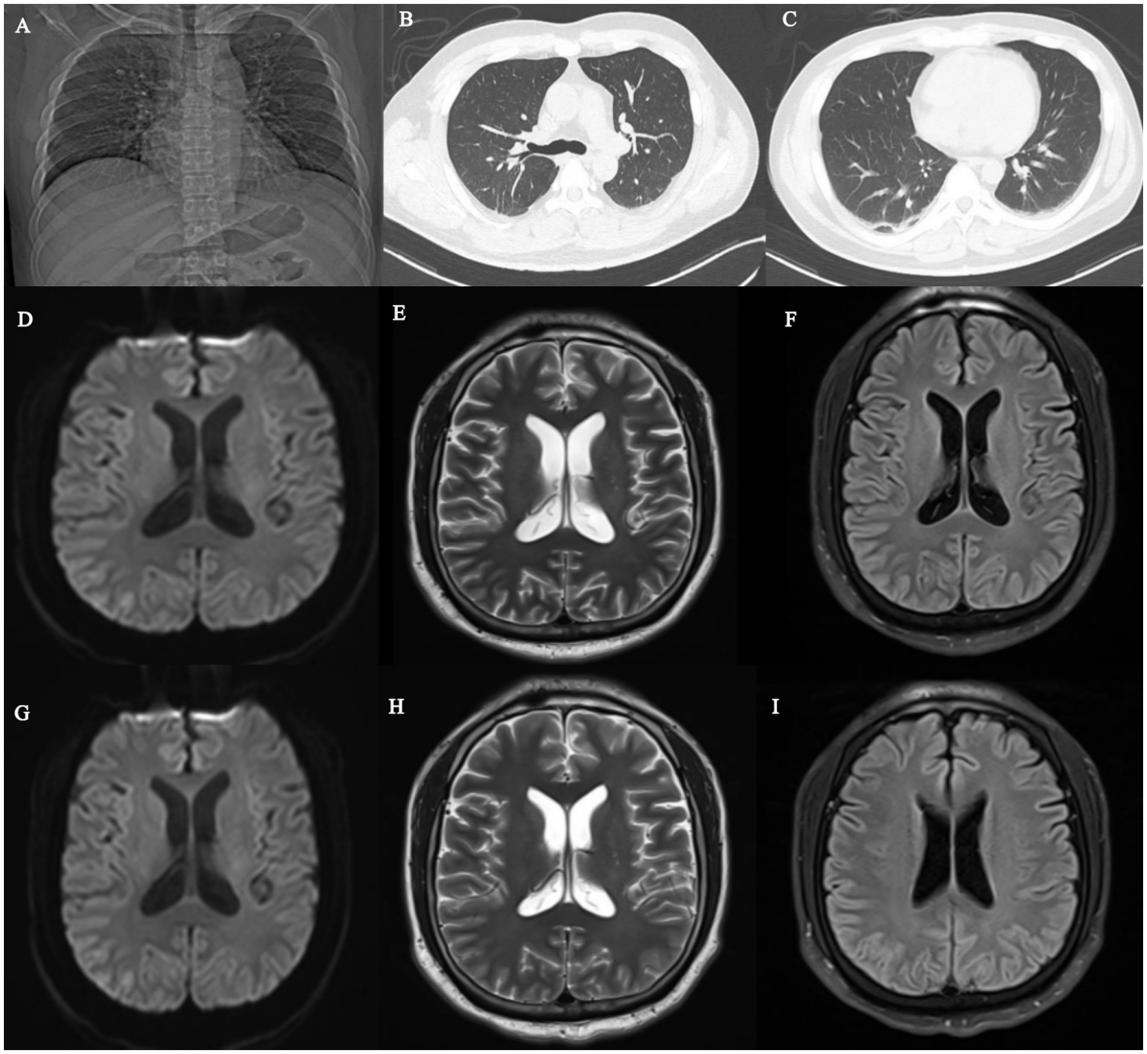
Comprehensive Radiological Findings. **(A)** Chest X-ray on the first admission showing no acute pulmonary infiltrates. **(B, C)** Chest CT on the second admission confirming the absence of typical intrapulmonary infiltrates suggestive of Legionella pneumonia. **(D-F)** Head MRI on the day of admission demonstrating no structural lesions or restricted diffusion on DWI, T2WI, and FLAIR sequences. **(G-I)** Follow-up head MRI prior to discharge remaining unremarkable, lacking the reversible splenial lesions occasionally associated with Legionnaires' encephalopathy.

He was admitted to the emergency department due to impaired consciousness. The patient developed fever symptoms 2 weeks prior to admission. The initial fever reached 38.5 °C (101.3 °F), accompanied by systemic symptoms including muscle aches in the limbs and fatigue, but without respiratory symptoms such as cough or sputum production. The patient presented to a local community health center, and a chest X-ray showed no evidence of acute infection (Figure 1). He was subsequently admitted to the General Internal Medicine Ward. Respiratory pathogen nucleic acid testing was negative for 11 respiratory viruses, including influenza virus and novel coronavirus, as well as eight bacterial pathogens. Complete blood count results showed a total white blood cell count of 2.67 × 10^9^/L and a neutrophil count of 1.42 × 10^9^/L. C-reactive protein was 9.35 mg/L, serum amyloid A (SAA) was 65.5 mg/L, procalcitonin was 0.67 mg/L, and interleukin-6 was 12.7 pg/ml. Based on the persistent high fever and to cover potential severe or atypical bacterial infections, the local physicians administered empirical broad-spectrum antimicrobial therapy. The regimen consisted of piperacillin-tazobactam 4.5 g intravenously every 8 h for 14 days, combined with azithromycin 0.5 g once daily for 3 days. The patient experienced a persistent fever for 10 days during hospitalization, with peak temperatures ranging between 39.5 and 40.0 °C. On the 11th day of hospitalization, the patient's fever resolved without recurrence. However, the patient developed drowsiness, with no abnormalities detected in cognitive function or limb motor function. Despite persistent drowsiness, the patient was discharged on the 14th day and returned home due to the approaching Spring Festival. The final discharge diagnosis given at this level was infectious fever of unknown pathogen.

On the day of discharge, the patient was found by family members to be extremely agitated, unresponsive to verbal stimuli, and exhibiting involuntary limb movements. There were no accompanying symptoms such as vomiting or seizures. Family members immediately transported the patient to our hospital's emergency department. Emergency cranial CT scan revealed no abnormalities. Upon admission, the patient was hemodynamically stable but exhibited extreme agitation and delirium (RASS score 4–5), rendering standard GCS assessment unfeasible. Meningeal irritation signs were absent, bilateral pathological signs were not elicited, and muscle tone in all limbs was normal. Bilateral pupillary light reflexes were sluggish, accompanied by slow horizontal nystagmus.

Given the patient had a recent history of a prolonged infectious fever of unknown pathogen before admission and later developed progressive impaired consciousness after the onset of drowsiness, we initially suspected a severe intracranial infection. Since the causative pathogen was unknown, we started empirical antimicrobial therapy with acyclovir (10 mg/kg q8 h) and ceftriaxone (3.0 g qd). Furthermore, the patient exhibited rapid progression of neurological symptoms combined with bilateral horizontal nystagmus, suggesting focal neurological impairment. Glucocorticoid therapy (methylprednisolone 80 mg q12 h) was added as adjuvant treatment. Concurrently, a combined sedation regimen of propofol and midazolam was administered to maintain the RASS score between 0 and 1. Basic supportive care, including nutritional support and anticoagulation, was also provided.

On admission, a lumbar puncture and CSF analysis were performed. Cranial MRI (plain and contrast-enhanced), MRA, and MRV studies showed no abnormalities (Figure 1). Chest CT revealed no inflammatory infiltrates (Figure 1). 3 days later, cerebrospinal fluid (CSF) metagenomic next-generation sequencing (mNGS) was negative; however, whole-blood targeted next-generation sequencing (tNGS) was subsequently performed as an adjunctive broad molecular test. Importantly, the tNGS assay used in this case was a broad-spectrum syndromic panel targeting more than 100 core pathogens associated with severe systemic and central nervous system infections, including Mycobacterium tuberculosis, Neisseria meningitidis, and Streptococcus pneumoniae. The panel identified Legionella pneumophila with a normalized sequence read count of 263, while no other clinically significant bacterial pathogen, including M. tuberculosis, was detected above the reporting threshold. Together with the clinical presentation, CSF profile, and neuroimaging findings, these results supported the diagnosis of Legionella pneumophila-associated encephalopathy and made alternative infectious etiologies with overlapping clinical features less likely. Laboratory and CSF findings from this admission are summarized in Tables 1, 2. A summary of the whole blood tNGS results is shown in Table 3. Based on these definitive molecular findings and the clinical presentation, the patient was diagnosed with Legionella pneumophila encephalopathy. The antimicrobial regimen was adjusted to levofloxacin (0.5 g once daily), while the glucocorticoid dosage remained unchanged. By the 7th day of hospitalization, the patient regained clear consciousness with normal cognitive function and limb mobility. The dose of glucocorticoid was subsequently reduced to 40 mg every 12 h. Follow-up cranial MRI remained unremarkable (Figure 1). The patient was successfully discharged on the 8th day of hospitalization. Following discharge, the patient was advised to continue taking oral levofloxacin 0.5 g once daily for a total of 3 weeks. Subsequent follow-up revealed full return to normal work status with no residual neurological sequelae.

**Table 1:** Laboratory examinations on first admission and second admission.

Laboratory Tests	First admission	Second admission
Median blood counts		
Leukocytes, × 10^9^/L	2.67↓(3.5–9.5)	12.30↑(3.5–9.5)
Neutrophils, × 10^9^/L	1.42↓(1.8–6.3)	9.47↑(1.8–6.3)
Lymphocytes, × 10^9^/L	1.1(1.1–3.2)	2.23(1.1–3.2)
Hemoglobin, g/L	146(130–175)	143(130–175)
Platelets, × 10^9^/L	182(125–350)	224(125–350)
Main biochemical parameters		
Creatinine, μmol/L	87(51–97)	66(51–97)
Urea, mmol/L	5.5(3.1–8.0)	4.3(3.1–8.0)
C-reactive protein, mg/L	9.35↑(< 5.0)	4.8(< 5.0)
PCT, mg/L	0.67↑(< 0.5)	< 0.1(< 0.5)
Interleukin-6, pg/mL	12.7↑(< 6.4)	4.78( ≤ 7)
Serum Amyloid A, mg/L	65.5↑(0–10)	6.82(0–10)
Bilirubin, μmol/L	12.1( ≤ 26)	22.2(0–23.0)
AST, U/L	31(9–50)	26(0–40)
ALT, U/L	38(15–40)	77(0–50)
LDH, U/L	390↑(120–250)	307↑(120–250)
Na, mmol/L	140(137–147)	135(132–147)

ALT, alanine aminotransferase; AST, aspartate aminotransferase; LDH, lactate dehydrogenase.

**Table 2:** Cerebrospinal fluid-related examinations.

Routine examination	Colorless and transparent
Characteristics	
Pressure, mmH_2_O	160 (70–180)
Red blood cell (RBC) count, 10^12^/L	0.0000
Nucleated cell count, 10^9^/L	0.045
Monocyte ratio, %	97
Polymorphonuclear cell ratio, %	3
Qualitative Pandy reaction	++(-)
Biochemistry	
Glucose	6.04↑(2.5–4.4)
Protein	1818.10↑↑(150–400)
Chloride	126.8(120–132)
Microbiological examination	
Culture	—
Gram staining	—
Metagenomic next-generation sequencing	—

**Table 3:** Summary of Peripheral Blood Targeted Next-Generation Sequencing (tNGS) Results.

Microbial category	Detected pathogens	Sequence read count	Clinical interpretation
Pathogenic microorganisms	Legionella pneumophila	263	Confirmed causative pathogen
Opportunistic pathogenic microorganisms	Negative	0	Ruled out opportunistic coinfection
Common human commensals	Negative	0	Background noise; not clinically significant

tNGS performed using the Illumina NextSeq 550Dx sequencing platform.

## Discussion

Legionella widely exists in natural and man-made aquatic environments, and human infection mainly occurs via inhalation of contaminated aerosols. Common environmental sources include cooling towers, central water supply systems, domestic hot water systems, showers, and humidifiers, especially during winter heating seasons. Indoor humidifiers can easily form aerosolized water droplets and provide suitable conditions for Legionella proliferation. In this case, the patient resided in an urban household and used a humidifier during the winter heating period, which represented a potential and plausible exposure source for Legionella infection. Although targeted environmental sampling and detection were not performed after diagnosis, this winter humidifier use was highly consistent with community-acquired, environmentally transmitted Legionella infection.

Severe Legionella infections advance swiftly, with fatality rates in intensive care units ranging from 10%−40% (1), which may lead to enduring neurological complications. This emphasizes to doctors the importance of early identification of Legionella encephalopathy in individuals lacking characteristic Legionella pulmonary symptoms.

Legionella pneumophila is a pathogen highly adapted to intracellular survival. Upon infecting humans, its primary target cells are alveolar macrophages and peripheral blood mononuclear cells (7). More than 86.3% of patients exhibit no direct evidence of Legionella infection in CSF examination (6). Although its primary site of infection and replication is in the lungs, Legionella bacteria can cause localized tissue damage, intense inflammatory responses, and the release of various toxic substances. These effects can traverse the blood-brain barrier (BBB) via the systemic circulation, exerting catastrophic distant effects on the central nervous system (6, 8, 9). In this case, both the regular cerebrospinal fluid test and the advanced metagenomic next-generation sequencing found no signs of infection, and the only result was a very high level of protein. This clinical presentation, devoid of direct pathogen colonization, coupled with the patient's swift response to high-dose glucocorticoids, robustly indicates that the encephalopathy stemmed from secondary neurological damage mediated by an “immune cytokine storm and toxins,” rather than direct bacterial-induced suppurative destruction.

The clinical presentation of Legionnaires' encephalopathy exhibits considerable variability and heterogeneity among patients (5). Neurological symptoms typically manifest during the acute phase of pneumonia following the appearance of pulmonary infiltrates. However, in this case, the patient developed an insidious onset after normalizing body temperature and in the absence of radiographic evidence of pneumonia. Concurrently, profound delirium and ocular motor abnormalities such as bilateral horizontal nystagmus strongly suggest a highly characteristic focal neurological impairment. In recent years, a highly specific radiological phenotype has frequently emerged in some Legionnaires' encephalopathy patients: encephalopathy with reversible corpus callosum commissural lesions. This manifests as an isolated, high-signal area located in the commissural region of the corpus callosum, strongly suggesting the presence of localized cytotoxic edema or edema caused by intra-myelin fluid retention (10, 11). Nonetheless, the patient's cranial MRI appeared normal, indicating that neurotoxic damage at the microscopic biochemical level may occur prior to observable macroscopic radiological structural alterations. Consequently, a standard MRI result should not exclude the diagnosis. Admission was principally characterized by altered awareness and the absence of normal respiratory symptoms, leading to the omission of standard urine Legionella antigen testing in the early stages. Furthermore, while serological testing (detection of Legionella antibodies) serves as a valuable diagnostic tool—particularly in advanced stages of infection where seroconversion has typically occurred (2–4 weeks post-onset)—it was not routinely performed in this case. The rapid and definitive identification of the pathogen via whole-blood tNGS, coupled with the patient's swift response to targeted therapy, precluded the necessity for retrospective serological confirmation. Nevertheless, this highlights that in delayed or advanced atypical presentations, clinicians should consider a multimodal diagnostic approach combining molecular screening (tNGS), urine antigens, and serology to prevent missed diagnoses.

According to authoritative guidelines from the Infectious Diseases Society of America and the American Thoracic Society (IDSA/ATS), therapeutic agents selected for Legionella infections with severe central nervous system complications must possess both excellent macrophage penetration and good BBB penetration. The preferred drugs are respiratory quinolones (such as levofloxacin), macrolides (such as azithromycin), and tetracyclines (such as doxycycline) (12, 13). Antibiotic treatment for immunocompetent adults with Legionella encephalitis should be given for at least 10–14 days, according to the guidelines. For those with weakened immune systems, such as organ transplant recipients or long-term steroid users, or in severe cases requiring intensive care and intubation, the antimicrobial treatment should be extended to 21 days to ensure complete elimination of the bacteria (5, 14). The patient discontinued azithromycin after only 3 days of treatment at the community hospital. Inadequate initial antibiotic therapy is considered the cause of bacterial persistence, subsequent toxin production, and the resulting encephalopathy observed in the later stages of the disease.

Experts recommend using high-dose glucocorticoids for a short time in patients with severe encephalopathy, but only if they are also receiving appropriate antimicrobial treatment (15). In this instance, because of the patient's profound awareness impairment and critical state, we commenced methylprednisolone pulse therapy before pathogen confirmation to mitigate the severe encephalitic response. The conclusive diagnosis of “non-purulent, immune-mediated mechanism” signifies that this atypical early steroid injection did not result in the dissemination of infection. Instead, it effectively interrupted the neurotoxic cascade prior to sufficient levofloxacin coverage, markedly enhancing the patient's prognosis.

A notable limitation of this report is the absence of environmental microbiological sampling. Although our epidemiological interview identified the uncleaned domestic air humidifier as the highly probable source of aerosolized infection, we did not retroactively culture the humidifier water. Given the patient's rapid neurological recovery following administration of glucocorticoids and targeted antibiotics, a formal environmental investigation was not conducted before discharge. Ideally, future atypical cases should pair clinical molecular diagnostics with immediate environmental sampling to definitively confirm the transmission vector.

### Advantages and limitations

This case report has several notable strengths. First, it describes an extremely rare phenotype of Legionella pneumophila encephalopathy without any respiratory symptoms, pulmonary infiltrates, or abnormal brain MRI findings, which can significantly improve clinicians' awareness of such atypical presentations. Second, it highlights the critical value of whole-blood tNGS in rapid etiological diagnosis for unexplained encephalopathy when conventional tests are negative. Third, it supports the benefit of early glucocorticoid use combined with targeted antibiotics in reversing severe immune-mediated neurological damage.

The main limitations of this report should also be acknowledged. As a single case report, the findings cannot be generalized to a broader population. In addition, Legionella serological testing and environmental sampling for source tracking were not performed, which limits further epidemiological and immunological analysis. Moreover, the exact mechanism of immune-mediated neurotoxicity in Legionella encephalopathy requires further basic and clinical research to verify.

## Conclusion

We outline an unusual presentation of Legionella encephalopathy occurring without the classic respiratory signs, lung infiltrates, or MRI lesions. Because the cerebrospinal fluid remained aseptic and the neurological deficits reversed rapidly following steroid administration, the underlying mechanism is highly likely driven by immune cascades and toxins rather than direct bacterial invasion. Ultimately, this scenario demonstrates that clinicians should not rule out Legionella simply because pneumonia is absent. Early application of molecular diagnostics is essential for catching such atypical neurotoxic manifestations.

### Clinical takeaways

- Legionella encephalopathy may present as an atypical, non-pneumonic form with normal imaging.
- Early pathogenic detection (blood tNGS or urine antigen) should be performed in unexplained acute encephalopathy.
- Targeted antibiotics plus early glucocorticoids can effectively improve neurological prognosis.

## Data Availability

The original contributions presented in the study are included in the article/supplementary material, further inquiries can be directed to the corresponding author.
